# The Epstein-Barr virus-miRNA-BART6-5p regulates TGF-β/SMAD4 pathway to induce glycolysis and enhance proliferation and metastasis of gastric cancer cells

**DOI:** 10.32604/or.2024.046679

**Published:** 2024-04-23

**Authors:** XUHUI ZHAO, XIAOMIN HUANG, CHUNYAN DANG, XIA WANG, YUJIAO QI, HONGLING LI

**Affiliations:** 1 The First Clinical Medical College, Gansu University of Traditional Chinese Medicine, Lanzhou, 730000, China; 2 Department of Oncology, Gansu People’s Hospital, Lanzhou, 730000, China; 3 The Clinical Medical College, Ningxia Medical University, Ningxia, 750004, China

**Keywords:** EBV, TGF-β/SMAD4, Glycolysis, Gastric cancer

## Abstract

**Background:**

EBV-miR-BARTs exhibit significant relevance in epithelial tumors, particularly in EBV-associated gastric and nasopharyngeal cancers. However, their specific mechanisms in the initiation and progression of gastric cancer remain insufficiently explored.

**Material and Methods:**

Initially, EBV-miRNA-BART6-5p and its target gene SMAD4 expression were assessed in EBV-associated gastric cancer tissues and cell lines. Subsequent transfection induced overexpression of EBV-miRNA-BART6-5p in AGS and MKN-45, and downregulation in EBV-positive cells (SUN-719). The subsequent evaluation aimed to observe their impact on gastric cancer cell proliferation, migration, and glycolytic processes, with the TGF-β/SMAD4 signaling pathway value clarified using a TGF-β inhibitor.

**Results:**

EBV-miRNA-BART6-5p exhibits pronounced upregulation in EBV-associated gastric cancer tissues and EBV-positive cells, while its target gene SMAD4 demonstrates downregulated expression. Upregulation of it can promote the proliferation and migration of gastric cancer cells. Additionally, We found EBV-miRNA-BART6-5p promotes glycolysis of gastric cancer cells. Inhibition of the TGF-β/SMAD4 signaling pathway resulted in suppressed proliferation and migration of gastric cancer cells, concomitant with a diminished glycolytic capacity.

**Conclusion:**

In this study, we found that EBV-miRNA-BART6-5p can target SMAD4, effectively increasing glycolysis in gastric cancer cells by regulating the TGF-β/SMAD4 signaling pathway, thereby enhancing the proliferation and metastasis of gastric cancer cells. Our findings may offer new insights into the metabolic aspects of gastric cancer.

## Introduction

Gastric cancer is among the most prevalent malignancies of the digestive system, ranking as the fourth most common cancer globally and the second leading cause of cancer-related deaths [1]. The 2014 TCGA database classified gastric cancer into four subtypes based on molecular typing and clinical features: EBV-associated, microsatellite instability (MSI), genome-stable (GS), and chromosomal instability (CIN) [2,3]. EBV-associated gastric carcinoma constitutes around 10% of all gastric carcinomas, exhibiting distinctive clinicopathological characteristics, such as male predominance, a relatively favorable prognosis, and localization in the gastric cardia with varying degrees of lymphoid infiltration [4–6]. Therefore, exploring potential targets of EBV-associated gastric carcinoma (EBVaGC) may represent a new avenue for treating patients with gastric cancer (GC).

As a DNA virus, EBV can encode various miRNAs that interact with host genes, forming a complex regulatory network that plays a crucial role in the onset and development of EBV-associated gastric cancer. Currently, EBV miRNAs consist of two major classes: BHRF1 and BART miRNAs [7,8], with BHRF1 RNAs located in the introns of BHRF and BART miRNAs situated in the introns of BART transcripts. In EBVaGC, 99% of virus-derived polynucleotide transcripts originate from BARTs. In recent years, several studies have explored the expression of BART miRNAs in EBVaGC compared to other tumors [9,10]. The results indicate that about 15% of BART miRNAs are found in EBVaGC, showing significantly higher expression levels than in lymphomas [11]. Naseem et al. [12,13] identified high expression levels of miRNA-BART 1-3p, 3, 4, 5-5p, 17-5p, 7-3p, 9-3p, 10-3p, 18-5p, and 2-5p in tissue samples from EBVaGC patients. Evidence suggests that EBV miRNAs play crucial roles in various biological processes in EBVaGC, including host immune evasion, latent infection, cell proliferation, apoptosis, and metastasis [14–17]. Thus, it is evident that EBV miRNA BARTs can promote the proliferation and invasive ability of gastric cancer cells. However, the mechanisms through which they regulate downstream signaling molecules remain unclear, necessitating further studies for a more detailed exploration.

SMAD4 is a specific tumor suppressor gene in pancreatic cancer [18,19], and the loss of SMAD4 function is significantly correlated with tumor invasion, poor progression, and poor prognosis [20–22]. SMAD4 has been reported to be involved in the development of glycolysis in pancreatic, colon, and breast cancers [23,24]. However, the mechanism by which SMAD4 functions in EBVaGC cells has not yet been elucidated. In our study, we observed a significant downregulation of SMAD4 in EBVaGC tissues and cell lines. Subsequently, we investigated whether SMAD4 is regulated by EBV miRNA-BART6-5p, thereby influencing the cell phenotype and glycolytic processes of gastric cancer cells. In our study, we observed a significant downregulation of SMAD4 in EBVaGC tissues and EBV-positive cell line. Subsequently, we investigated whether SMAD4 is regulated by EBV miRNA-BART6-5p, thereby influencing the cell phenotype and glycolytic processes of gastric cancer cells.

## Materials and Methods

### Cell culture and reagents

AGS and MKN-45 are EBV-negative cell lines, while SUN719 is an EBV-positive cell line. Gastric cancer cells were cultured in RPMI-1640 supplemented with 10% fetal bovine serum (FBS) and 1% penicillin-streptomycin. The human embryonic kidney cell line HEK293T was cultured in Dulbecco’s Modified Eagle’s Medium (DMEM) with 10% fetal bovine serum (FBS) and 1% penicillin-streptomycin. All cells were maintained in a 37°C incubator with 5% CO_2_.AGS and MKN-45 cells were given free of charge by the laboratory of Gansu University of Traditional Chinese Medicine; SUN719 cells were purchased from Zhejiang Meisen Cell Technology Co. (China).

### Patients and tissue specimens

Cancer tissues and corresponding adjacent normal tissues were collected from three patients with gastric cancer diagnosed pathologically in Gansu Provincial People’s Hospital. Pathological assessment was performed by reviewing archived HE sections, immunohistochemical sections and Epstein-Barr virus-encoded small RNA (EBER) *in situ* hybridisation sections. All enrolled cases were independently reviewed and confirmed by an experienced pathologist specialising in gastric cancer. This study was approved by the Medical Ethics Committee of Gansu Provincial People’s Hospital and complied with the ethical requirements of the Helsinki Declaration (Approval No. 2023-171). The written informed consent was obtained from all participants.

### Bioinformatics analysis and dual luciferase reporter gene assay

Two publicly available prediction algorithms, RepTar (http://reptar.ekmd.huji.ac.il/) and DIANA TOOLS (http://diana.imis.athena-innovation.gr/DianaTools/index.php?r=tarbase/index), were used to predict potential target genes for EBV-miR-BART6-5p. The predicted candidate targets were then confirmed with low minimum free energy (MFE) using RNAhybrid software (https://bibiserv.cebitec.uni-bielefeld.de/). To determine if SMAD4 is a direct target of EBV-miR-BART6-5p, wild-type or mutant SMAD4 luciferase reporter vectors were transfected into HEK293T cells with EBV-miR-BART6-5p or normal controls. Firefly and Renilla luciferase activity were measured 48 h post-transfection using the Dual Luciferase Reporter Assay System. All measurements were performed in triplicate.

### Cell proliferation, colony formation assays, and EdU cell proliferation assay

For the cell proliferation assay, gastric cancer cells were seeded into 96-well plates at a density of 1500 cells per well and cultured in a 37°C cell culture incubator with 5% CO_2_ for 24, 48, and 72 h. Subsequently, 10 μL Cell Counting Kit-8 solution was added to each well and incubated for 2 h, and the absorbance value (OD) was measured at 450 nm. For the colony formation assay, gastric cancer cells were plated in 6-well plates at a density of 1000 cells per well and cultured in a 37°C cell culture incubator with 5% CO_2_ for 14 days. Once a single colony contained more than 50 cells, as counted under a microscope, the colonies were washed three times with PBS and stained with crystal violet staining solution. For the colony formation assay, gastric cancer cells were plated in 6-well plates at a density of 1000 cells per well and cultured in a 37°C cell culture incubator with 5% CO_2_ for 14 days. Once a single colony contained more than 50 cells, as counted under a microscope, the colonies were washed three times with PBS and stained with crystal violet staining solution.

### Wound healing assay and transwell migration assay

To investigate the effect of EBV-miR-BART6-5p on cell migration, we employed wound healing assay and Transwell migration assay. For the wound healing assay, gastric cancer cells were plated in 6-well plates at a density of 10 × 10^5^ in a 37°C cell culture incubator containing 5% CO_2_. When the cell confluence reached 90%–95%, a wound was created through the confluent monolayer using a 200 μL sterile pipette tip. After washing twice with PBS to remove cellular debris, photographs were taken under the microscope at 0 h, and after 24 h, additional photographs were taken to observe the degree of wound healing. For the Transwell migration assay, gastric cancer cells were seeded at a density of 1 × 10^4^ in 8.0 μm pore size Transwell chambers (Corning Life Sciences, Acton, MA, USA) in serum-free medium. A total of 700 μL of 1640 medium containing 10% FBS was added to the lower chamber. The chambers were incubated in a 37°C cell culture incubator with 5% CO_2_ for 24 h. Subsequently, the upper chambers were removed, fixed with 4% paraformaldehyde for 20 min, stained with 0.1% crystal violet for 15 min, and gently wiped with a cotton swab. Photographs were then taken under a microscope to assess the migration level of each group of transfected cells, and the wounding area was evaluated using ImageJ software. The experiment was repeated three times.

### Quantitative reverse transcription PCR (qRT-PCR)

Total RNA was extracted from the cells using Trizol reagent (Invitrogen), and cDNA was synthesized using the MonScript RTIII All-in-One Mix with dsDNase kit (Monad Biotech Co., Ltd.). PCR analysis was conducted using the SYBR Green qPCR Mix Kit (Monad Biotech Co., Ltd.) following the manufacturer’s instructions. The sequences of the primers were 5′-CGTCAGCTGTCCGAGTAGAGGTAAGGTTGGTCC AAT-3′ and 5′-TGTCAGGCAACCGTATTCACCCCTA TGG-3′ for EBV miR-BART6-5p, 5′-CAGCACTACCAC CTGGACTG-3′ and 5′-TGTCGATGACACTGACGCA A-3′ for SMAD4, 5′-TTG GAGCCACCACTCACCCTA-3′ and 5′-GAGCCCATTG TCCGTTACTTTC′-3′ for HK2, 5′-GCTTCTCCAACTGGACCTCAAA-3′ and 5′-GAAG AACAGAACCAGGAGCACAG′ for GLUT1 and 5′-ATGGGGAAGGTGAAGGTCG-3′ and 5′-GGGGG TCATTGATGGCAACAATA-3′ for GAPDH. GAPDH expression was used to specify miRNA and mRNA expression, respectively. The qRT-PCR reaction was repeated three times for each sample in three independent experiments. Relative quantification was used (2^-ΔΔCt^).

### Western blot analyses

Cell lysates were mixed with sample buffers and protease inhibitors in RIPA buffer, then heated at 100°C for 5 min. Abbreviations are expanded upon when first used. The language used is objective, concise, and technical terminology is consistent and accurate. After electrophoresis on an 8% SDS-polyacrylamide gel, the separated proteins were transferred to a polyvinylidene fluoride (PVDF) membrane (Millipore, Billerica, MA, USA). The membranes were sealed before probing with the following antibodies: rabbit anti-SMAD4 (1:1000; Proteintech, China), rabbit anti-GLUT1 (1:1000; Proteintech, China), rabbit anti-HK2 (1:1000; Proteintech, China), and rabbit anti-GAPDH (1:1000; Proteintech, China). The bound antibodies were detected using a horseradish peroxidase (HRP)-coupled anti-rabbit secondary antibody (Proteintech, China) at a dilution of 1:3000 for 1 h at room temperature. Protein bands were visualized using an enhanced chemiluminescence detection system (Amersham Bioscience, GE Healthcare, Piscataway, NJ, USA), and the membranes were exposed to X-ray film. The GAPDH antibody was used to confirm equal loading across gel lanes. The density of each protein band was then measured and quantified using ImageJ software.

### Transfection of EBV-miR-BART6-5p and miRNA Inhibitor

The EBV-miR-BART6-5p plasmid and control plasmid PCDH were generously provided by Fudan University. The EBV-miR-BART6-5p inhibitor and negative control inhibitor (inhibitor-NC) were purchased from RiboBio Biological Company (Guangzhou, China). Lipofectamine 3000 (Invitrogen, Carlsbad, California, USA) was used for all transfection experiments according to the manufacturer’s protocol. Protein and RNA were extracted 48 h after transfection.

### Statistical analysis

Statistical analysis was conducted using SPSS 19.0 and GraphPad Prism software (8.0.1). Analyses included *t*-tests for two groups, multiple one-way analysis of variance (ANOVA), and statistical significance was considered at *p* < 0.05. Single, double, and triple asterisks denote significance levels at *p < 0.05, **p < 0.01, and ***p < 0.001, respectively.

## Results

### High expression of EBV-miR-BART6-5p in gastric cancer tissues of EBVaGC patients, with SMAD4 exhibiting relatively low expression

**Figure 1:**
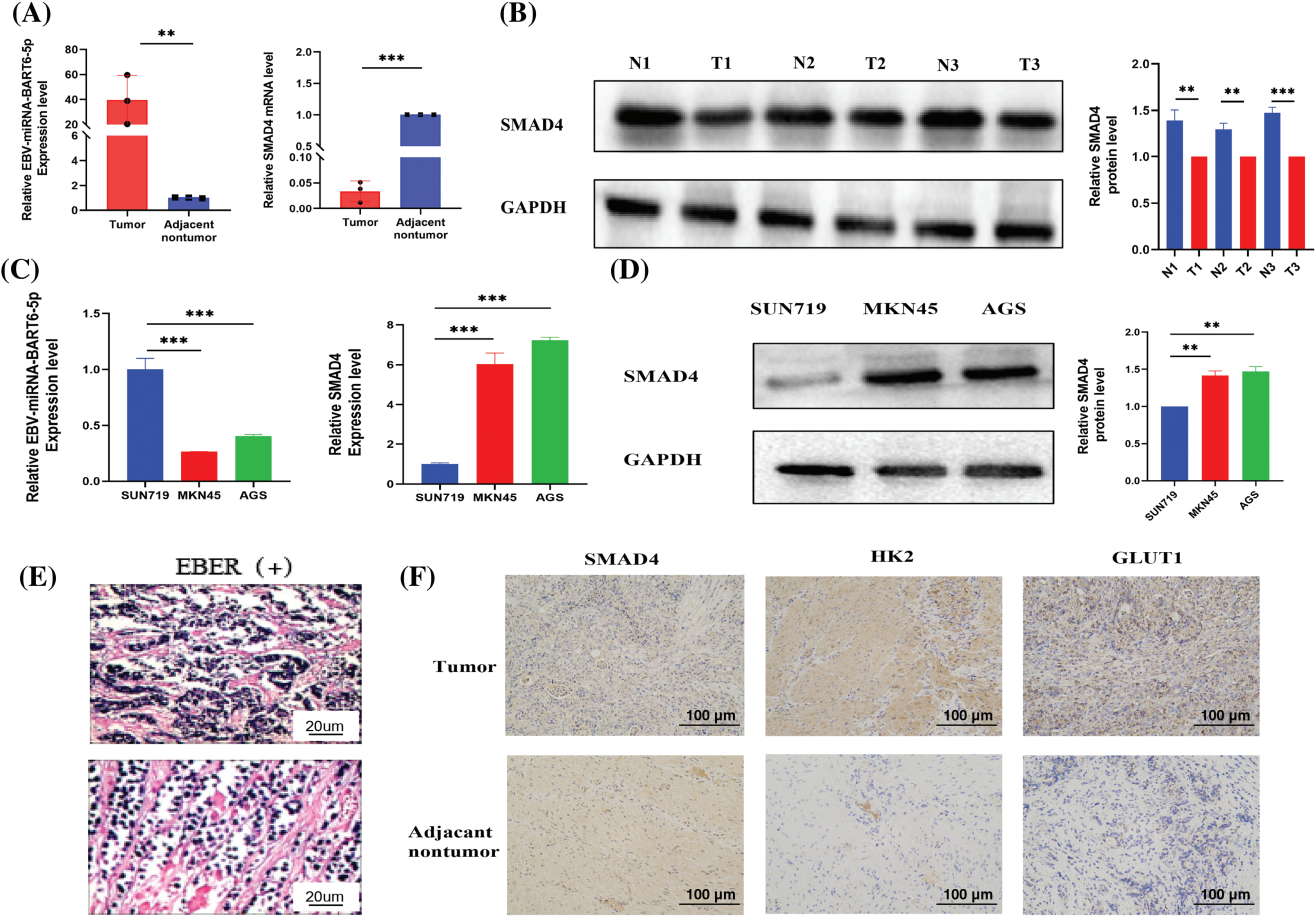
Expression of EBV-miR-BART6-5p and SMAD4 in Gastric Cancer Tissues and Cell Lines. (A) qRT-PCR measured the expression of EBV-miR-BART6-5p and SMAD4 mRNA in gastric cancer tissues and corresponding adjacent normal tissues. (B) Western blot assessed the protein expression of SMAD4 in gastric cancer tissues and adjacent normal tissues. (C) qRT-PCR determined the expression of EBV-miR-BART6-5p and SMAD4 mRNA in gastric cancer cell lines (SUN719, MKN45, and AGS). (D) Western blot evaluated the protein expression of SMAD4 in gastric cancer cell lines. (E–F) Positive EBER detection by *in situ* hybridization and IHC measured the protein expression of SMAD4, GLUT1, HK2 in gastric cancer tissues and adjacent normal tissues (200×). *p < 0.05, **p < 0.01; ***p < 0.001.

Three pairs of gastric cancer tissues from EBVaGC patients with Epstein-Barr virus-encoded small RNA (EBER) positive and their corresponding adjacent normal tissues, along with gastric cancer cells, were selected. qRT-PCR and Western blot analyses were conducted to assess the expression of EBV-miR-BART6-5p and the target gene SMAD4. The results indicated higher expression of EBV-miR-BART6-5p in gastric cancer tissues of EBVaGC patients and elevated levels in the SUN719 cell line compared to AGS and MKN45, with a relatively low expression of the target gene SMAD4 (Figs. 1A–1D). In addition, we found that SMAD4 was low-expressed in gastric cancer tissues and high-expressed in the corresponding adjacent normal tissues and GLUT1 and HK2, key proteins for glycolysis, are relatively more highly expressed in gastric cancer tissues (Figs. 1E, 1F). This suggests that EBV-miR-BART6-5p is upregulated and may act as a pro-oncogenic factor, while the downregulation of oncogene SMAD4 promotes gastric carcinogenesis.

### EBV-miR-BART6-5p targets SMAD4 gene, suppressing its expression in gastric cancer cells

**Figure 2:**
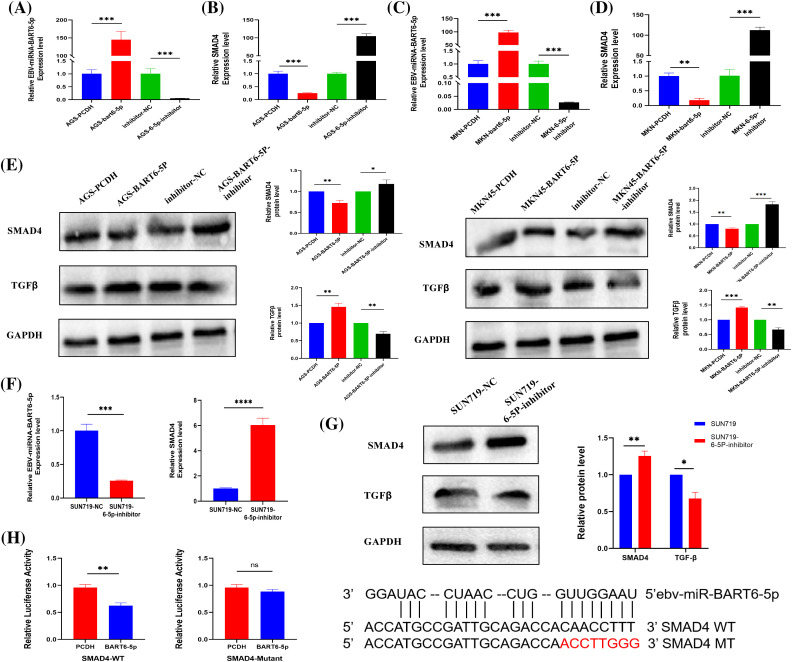
(A–D) The established EBV-miR-BART6-5p stably transfected cell line was tested for EBV-miR-BART6-5p expression levels and SMAD4 mRNA levels. The transfected BART6-5p-inhibitor was tested after Rescue experiments for EBV-miR-BART6-5p expression levels and mRNA levels of SMAD4. (E) Down-regulation of the protein level of the target gene SMAD4 after transfection with EBV-miR-BART6-5p. (F–G) The expression level of EBV-miR-BART6-5p can be inhibited, and the mRNA and protein levels of the target gene SMAD4 can be increased after transfection of the BEV-positive cell line SUN719 with the BART6-5p-inhibitor. (H) SMAD4 was demonstrated as a direct target of EBV-miR-BART6-5p using 293T cells by co-transfection with EBV-miR-BART6-5p and luciferase reporter genes containing either the wild-type (SMAD4-WT) or mutant (SMAD4-Mutant) in the 3′-UTR of SMAD4. The results showed that the EBV-miR-BART6-5p attenuated the luciferase activity of SMAD4-WT but not that of the SMAD4 mutant, suggesting that SMAD4 is a target gene of EBV-miR-BART6-5p. *p < 0.05, **p < 0.01; ***p < 0.001, ****p < 0.0001.

Based on bioinformatics analysis, we predicted that the SMAD4 gene might be regulated by EBV-miR-BART6-5p. Our experimental results demonstrated that EBV-miR-BART6-5p significantly inhibited the expression of SMAD4. We transfected the EBV-miR-BART6-5p plasmid into two EBV-negative gastric cancer cell lines, AGS and MKN-45. Confirmation was achieved through real-time fluorescence quantitative PCR and Western blot analysis (Figs. 2A, 2B). We observed a significant reduction in both mRNA and protein levels of SMAD4, along with elevated TGF-β expression in the stably transfected cell lines (Figs. 2C–2E).For rescue experiments, we added the EBV-miR-BART6-5p inhibitor to the stably transformed cell lines. We observed that down-regulation of EBV-miR-BART6-5p led to the re-regulation of SMAD4 expression at both mRNA and protein levels. However, TGF-β expression levels were decreased (Figs. 2A–2E). Furthermore, we inhibited endogenous EBV-miR-BART6-5p in the EBV-positive cell line SUN-719, inducing upregulation of SMAD4 at both the mRNA and protein levels (Figs. 2F–2G). To confirm SMAD4 as a direct target of EBV-miR-BART6-5p, we established dual luciferase reporter vectors containing either the wild-type (WT) binding sequence of EBV-miR-BART6-5p (SMAD4-WT) or a mutant of the SMAD4 3′-UTR (SMAD4 mutant). Co-transfection of the EBV-miR-BART6-5p plasmid and luciferase reporter vectors in 293T cells showed significant attenuation of luciferase activity for SMAD4-WT, while no effect was observed on SMAD4 mutants (Fig. 2H). The results above confirm that EBV-miR-BART6-5p directly targets SMAD4, inhibiting its expression in gastric cells.

### EBV-miR-BART6-5p promotes proliferation and migration of gastric cancer cells

**Figure 3:**
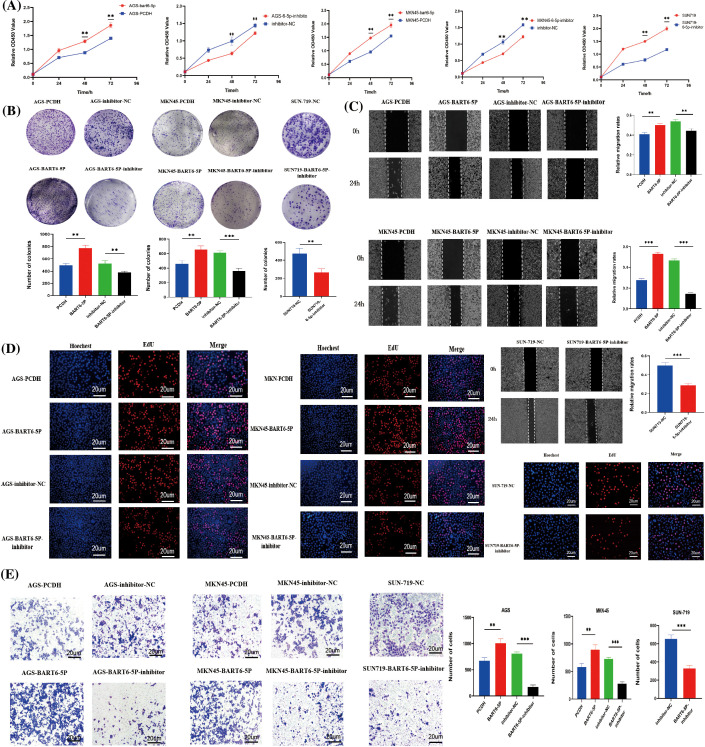
(A–E) EBV-miR-BART6-5p promotes proliferation and migration of gastric cancer cells. To evaluate the effect of BART6-5p on the proliferation and migration of gastric cancer cells, we transfected BART6-5p into AGS and MKN45 cells, in addition to BART6-5p inhibitor into the stable-transfer cell line and SUN719. The results of CCK8 and the representative images and quantification of the Colony formation, EdU proliferation, wound healing, and Transwell assays showed that BART6-5p overexpression could promote the proliferation and migration of gastric cancer cells (200×). However, when BART6-5p was inhibited, the proliferation and migration of gastric cancer cells were slowed down. Data are presented as the mean ± SD. *p < 0.05, **p < 0.01; ***p < 0.001.

To further investigate the function of EBV-miR-BART6-5p in gastric cancer cells, AGS and MKN-45 cells were transfected with EBV-miR-BART6-5p plasmid to establish EBV-miR-BART6-5p stably-transfected cell lines. CCK8, Colony formation, EdU proliferation, wound healing, and Transwell assays were used to measure the proliferative and migratory capacities of the transfected cells. The results showed that EBV-miR-BART6-5p significantly promoted the proliferation and migration gastric cancer cells. To determine whether the EBV-miR-BART6-5p inhibitor had an opposite function to the EBV-miR-BART6-5p, we inhibited endogenous EBV-miR-BART6-5p in SUN-719 and EBV-miR-BART6-5p inhibitor was added into EBV-miR-BART6-5p stably-transfected cell lines, and observed cell proliferation and migration. The results of the clone formation, CCK8, EdU proliferation, Transwell, and wound healing assays indicated that the EBV-miR-BART6-5p inhibitor reduced the proliferation and migration ability of SUN-719 cells (Figs. 3A–3E). These results demonstrate that EBV-miR-BART6-5p promotes proliferation and migration in gastric cancer cells.

### EBV-miR-BART6-5p promotes glycolysis of gastric cancer cells

**Figure 4:**
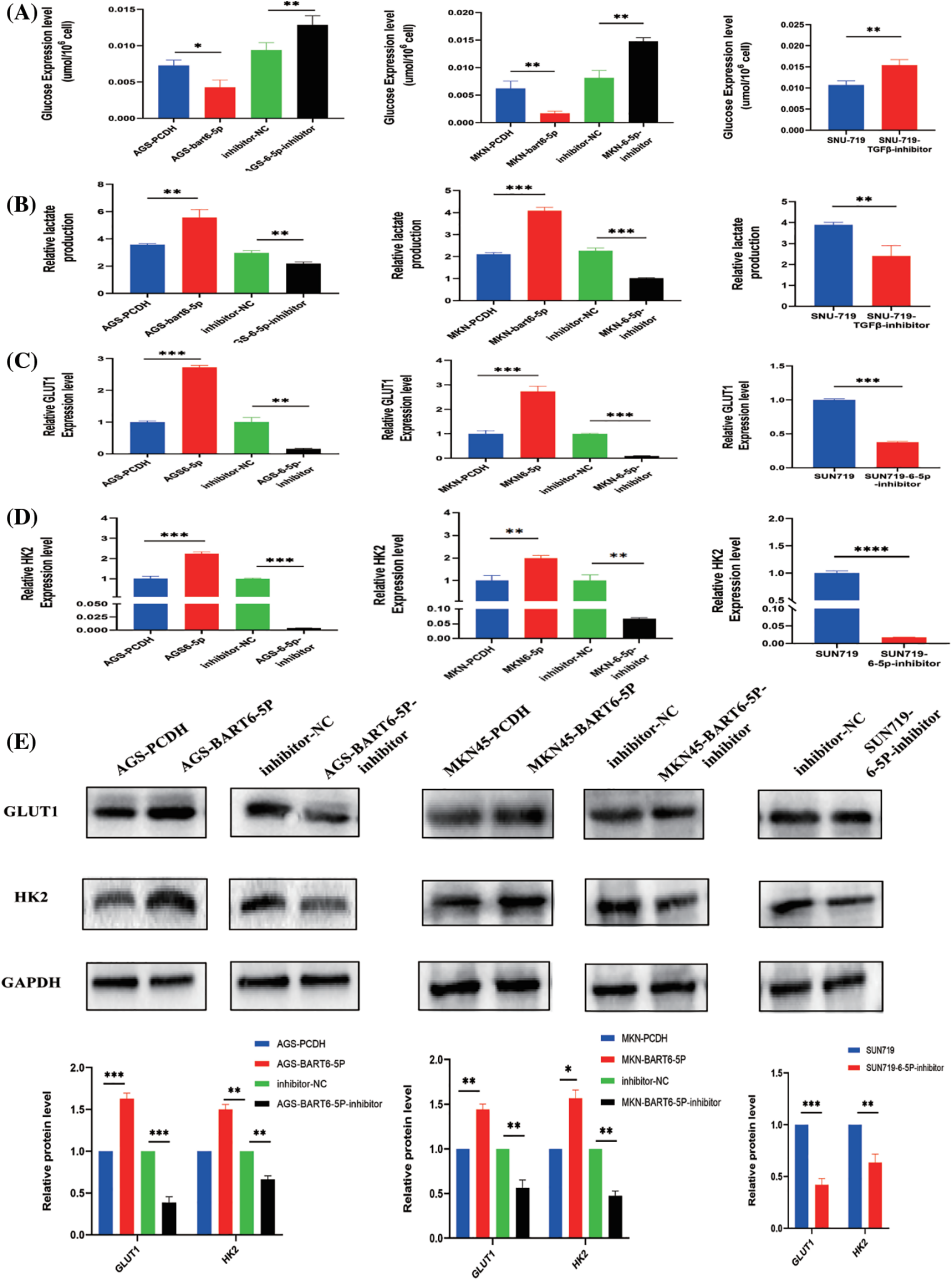
To investigate the regulatory role of EBV-miR-BART6-5p in the glycolytic process, we analyzed glucose and lactate levels in gastric cancer cell lines overexpressing or inhibiting BART6-5p. Overexpression of BART6-5p led to increased glucose consumption and excessive lactate accumulation, while inhibition of BART6-5p resulted in the opposite effect (A, B). Additionally, we examined key glycolysis-related molecules at the mRNA and protein levels. Overexpression of BART6-5p upregulated the expression of GLUT1 and HK2, whereas inhibition of BART6-5p downregulated their expression (C–E). *p < 0.05, **p < 0.01; ***p < 0.001, ****p < 0.0001.

During glycolysis, glucose is the initiating substance and lactate is the final product. To verify the role of EBV-miR- BART6-5p in gastric cancer glucose metabolism, we detected lactate production and glucose levels in the constructed EBV-miR-BART6-5p overexpressing stably-transfected gastric cancer cell lines. Following the simultaneous transfection of the BART6-5p-inhibitor to down-regulate the expression of BART6-5p in the SUN719 cell line and the stably-transformed cell line, we reassessed lactate production and glucose levels. In comparison to the control PCDH, the EBV-miR-BART6-5p overexpressing cell lines exhibited increased glucose consumption and higher levels of lactate production. Furthermore, compared to the inhibitor-NC group, the addition of the BART6-5p-inhibitor showed an opposing trend in glucose and lactate levels (Figs. 4A, 4B), and the silencing of BART6-5p reversed glucose consumption and lactate production. GLUT1 and HK2, essential enzymes in glucose metabolism, catalyze crucial steps in the glycolytic pathway.We assessed the mRNA and protein levels of GLUT1 and HK2, observing a significant increase in their expression with the overexpression of EBV-miR-BART6-5p. Conversely, silencing EBV-miR-BART6-5p led to a decrease in the expression of GLUT1 and HK2 (Figs. 4C–4E). Thus, our findings provide evidences that EBV-miR-BART6-5p promotes glycolysis in gastric cancer.

### EBV-miR-BART6-5p regulates phenotype and glycolysis processes of gastric cancer cells via the TGF-β/SMAD4 pathway

**Figure 5:**
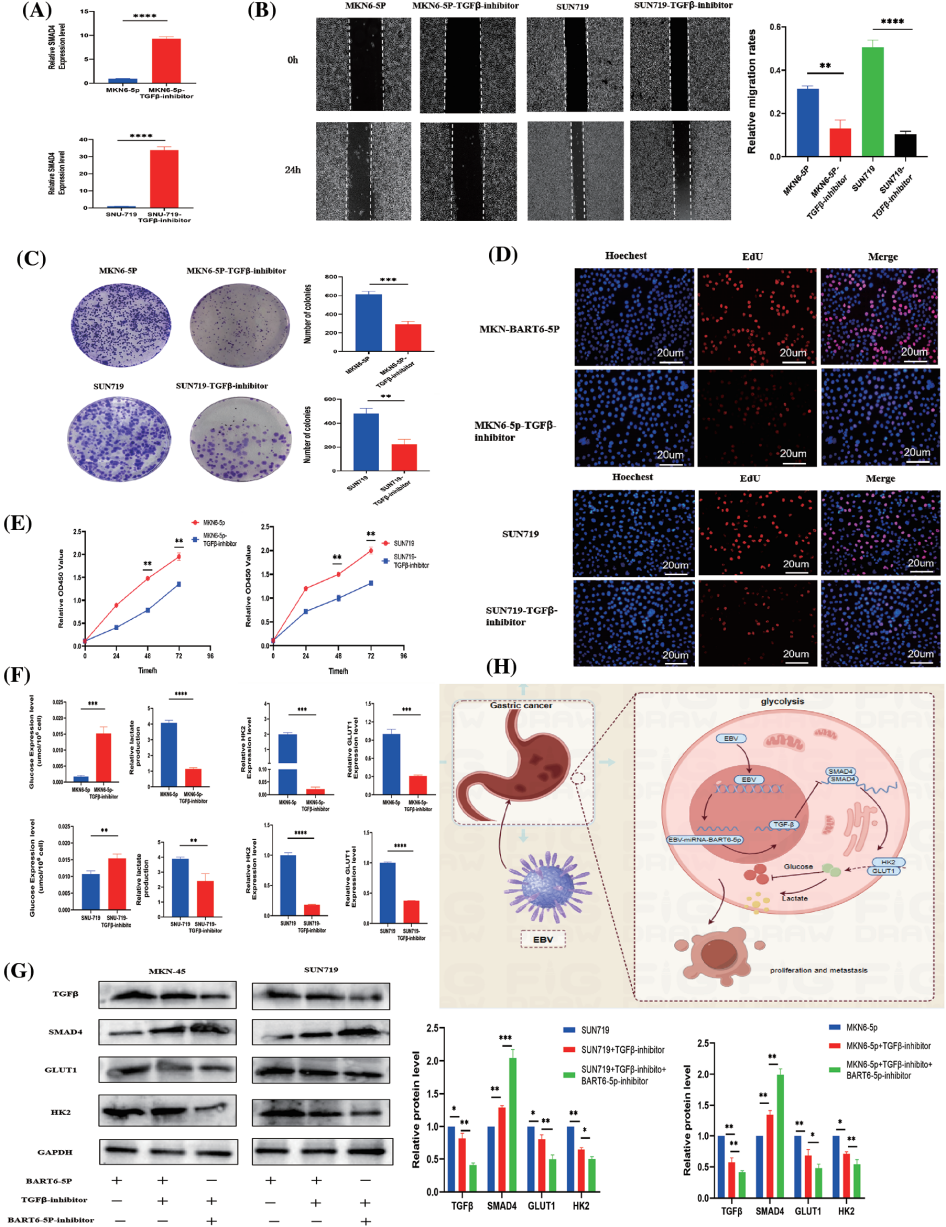
In investigating the regulatory influence of the TGF-β/SMAD4 pathway on the phenotype and glycolytic capability of gastric cancer, we examined cellular phenotype and glycolysis levels following the application of a TGF-β inhibitor. (A) The mRNA expression of SMAD4 was assessed following treatment with a TGF-β inhibitor, leading to a significant elevation in SMAD4 expression. (B) Wound-healing assays revealed a notable reduction in the migration capability of gastric cancer cells upon inhibition of the TGF-β signaling pathway. (C–E) Results from cell proliferation assays, including colony formation, CCK8, and EdU incorporation, suggested that the TGF-β signaling pathway influences the proliferative capacity of gastric cancer cells. (F–G) After inhibiting TGF-β signaling, there was a concurrent inhibition in glucose consumption, lactate production, and key glycolytic proteins, leading to a reduction in glycolytic capacity. (H) Schematic illustration summarizes that EBV-miR-BART6-5p regulates glycolysis processes of gastric cancer cells via the TGF-β/SMAD4 pathway. *p < 0.05, **p < 0.01; ***p < 0.001, ****p < 0.0001.

To elucidate the impact of EBV-miR-BART6-5p on gastric cancer cells, we explored its regulatory mechanisms within the TGF-β/SMAD4 pathway. The addition of a TGF-β inhibitor to EBV-miR-BART6-5p overexpressing stably-transformed gastric cancer cell lines and the EBV-positive cell line SUN719 revealed intriguing insights. Blocking TGF-β resulted in an upregulation of the target gene SMAD4 (Fig. 5A). Subsequent assessments, including colony formation, CCK8, EdU proliferation, and wound healing assays, demonstrated a deceleration in the proliferation and migration rates of gastric cancer cells (Figs. 5B–5E). Moreover, glucose consumption and lactate production were reversed upon TGF-β inhibition (Fig. 5F). The mRNA and protein levels of GLUT1 and HK2, pivotal enzymes in glucose metabolism, decreased, indicating a shift in glycolysis processes (Figs. 5F–5G). Furthermore, we found that the expression of TGF-β was further inhibited, as well as the expression of GLUT1 and HK2, after we added both TGF-β inhibitor and BART6-5p inhibitor to both cell lines, indicating that the level of glycolysis was obviously further attenuated. These observations collectively suggest that EBV-miR-BART6-5p exerts its regulatory effects on the phenotype and glycolysis of gastric cancer cells through modulation of the TGF-β/SMAD4 pathway.

## Discussion

EBV-associated gastric cancer (EBVaGC) is a distinct type of gastric cancer induced by the Epstein-Barr virus (EBV). EBV-encoded miRNAs are crucial in maintaining latent viral infection and fostering the malignant transformation of host cells. Despite this, research on EBV-encoded miRNAs in gastric cancer remains limited. In our study, we delved into the role of EBV-miRNA-BART6-5p in the development of gastric cancer. In the initial stages, we identified through bioinformatics that EBV-miRNA-BART6-5p could modulate the target gene SMAD4. Our investigation revealed a significant upregulation of BART6-5p expression in tissue specimens from EBVaGC patients, concurrently inhibiting the expression of the target gene SMAD4. Subsequently, we transfected the EBV-miRNA-BART6-5p plasmid into gastric cancer cells to observe its impact on cell phenotype. Our findings indicated that BART6-5p promotes the proliferation and migration of gastric cancer cells. Additionally, we observed that EBV-miRNA-BART6-5p regulates the target gene SMAD4, influencing the glycolysis process in gastric cancer cells.

Cancer cells exhibit a distinctive energy metabolism, characterized by increased glucose consumption and conversion of pyruvate to lactate, even in aerobic conditions, termed aerobic glycolysis [25,26]. This heightened glycolytic metabolism is a hallmark of tumor cells, providing a growth advantage through aberrantly elevated glucose uptake and lactate accumulation [27,28]. Previous reports indicate that EBV-miR-BART1 relies on glycolysis to influence HIF1α through PTEN, leading to the metastasis and dissemination of nasopharyngeal carcinoma cells [29]. Additionally, EBV-miR-BART1-5p activates the AMPK/mTOR/HIF1 pathway in nasopharyngeal carcinoma cells, promoting the aerobic glycolysis process [30]. However, the impact of EBV-miR-BART on glycolysis in gastric cancer has not been documented. Our investigation revealed that EBV-miRNA-BART6-5p influenced the levels of glucose and lactate, along with the mRNA and protein levels of GLUT1 and HK2, key enzymes in glucose metabolism [31,32]. Additionally, it modified the expression of TGF-β upstream of SMAD4. Thus, we identified that BART6-5p modulates the TGF-β/SMAD4 signaling pathway to promote glycolysis in gastric cancer cells.

SMAD4 markedly diminishes the rate of extracellular acidification and elevates the rate of oxygen consumption in cancer cells. Deletion of SMAD4 initiates a process of metabolic reprogramming. Specifically, the expression of SMAD4 significantly diminishes the rate of extracellular acidification and elevates the rate of oxygen consumption in pancreatic cancer, leading to metabolic reprogramming in this context. Knockdown of SMAD4 in pancreatic cancer cells enhances glycolysis and diminishes mitochondrial function [33,34]. The significance of SMAD4 deletion in tumorigenesis and metabolic reprogramming is notable [35,36]. The TGF-β/SMAD4 signaling pathway inhibits the expression of the glycolytic enzyme PGK1. In SMAD4 deletion-induced pancreatic cancers, there is an upregulation of PGK1 expression, leading to enhanced aerobic glycolysis and increased invasive behaviors [37]. In colon cancer, SMAD4 deficiency results in increased MMP9 expression in colon cancer cells, amplifying the expression of the hypoxia-inducible factor GLUT1, thereby promoting aerobic glycolysis processes [38]. In breast cancer, studies have demonstrated that SMAD4 promotes the ectopic expression of UCP2, triggering the glycolytic process and thereby promoting tumor growth [39–41]. SMAD4 specifically interacts with HIF1α under hypoxic conditions in various tumors, providing a molecular basis for the differential regulation of target genes [42]. Despite the known role of SMAD4 in regulating the onset of glycolytic processes in these tumors, its role in gastric cancer has been underexplored. In our study, we, for the first time, report that SMAD4, the target gene corresponding to EBV-miR-BART6-5p, influences tumorigenesis through glycolysis in gastric cancer. This study is expected to offer new perspectives for the treatment of EBVaGC.

Previous studies have demonstrated that EBV miRNAs can influence tumor development through various signaling pathways. Wong et al. reported that the upregulation of EBV-encoded miRNAs may modulate signaling pathways, including TGF-β, Wnt, and MAPK, which are closely associated with cell proliferation, migration, and cell cycle arrest [43]. Verhoeven et al. demonstrated that overexpression of EBV-miR-BART8-3p promotes EMT, invasion, and migration by activating the NF-κB and Erk38/1 signaling pathways, and directly targets RNF2 in NPC cells [44]. Our study demonstrates that the overexpression of EBV-miR-BART6-5p inhibits the expression of its target gene, SMAD4, thereby promoting the proliferation and migration of GC cells. This alteration also influences the expression of the TGF-β signaling pathway upstream of SMAD4, leading to changes in glucose consumption, increased lactate production, and an upregulation of GLUT1 and HK2 levels, ultimately driving glycolysis. In conclusion, this study elucidates the specific molecular mechanism of EBV-miRNA-BART6-5p associated with glycolysis in gastric carcinogenesis. This finding may potentially serve as a therapeutic target for gastric cancer treatment and contribute to novel ideas for the diagnosis and treatment of gastric cancer.

## Data Availability

The original contributions presented in the study are included in the article. Further inquiries can be directed to the corresponding author.
